# High proportion of tuberculosis transmission among social contacts in rural China: a 12-year prospective population-based genomic epidemiological study

**DOI:** 10.1080/22221751.2022.2112912

**Published:** 2022-08-31

**Authors:** Meng Li, Mingcheng Guo, Ying Peng, Qi Jiang, Lan Xia, Sheng Zhong, Yong Qiu, Xin Su, Shu Zhang, Chongguang Yang, Peierdun Mijiti, Qizhi Mao, Howard Takiff, Fabin Li, Chuang Chen, Qian Gao

**Affiliations:** a Key Laboratory of Medical Molecular Virology (MOE/NHC/CAMS), School of Basic Medical Science, Shanghai Medical College, Shanghai Institute of Infectious Disease and Biosecurity, Fudan University, Shanghai, People’s Republic of China; b National Clinical Research Center for Infectious Diseases, Shenzhen Third People's Hospital, Shenzhen, People’s Republic of China; c Wusheng County Center for Disease Control and Prevention, Guang’an, People’s Republic of China; d Heilongjiang Provincial Center for Tuberculosis Prevention and Control, Harbin, People’s Republic of China; e School of Public Health, Wuhan University, Wuhan, People’s Republic of China; f Institution for Tuberculosis Prevention and Control, Sichuan Provincial Center for Disease Control and Prevention, Chengdu, People’s Republic of China; g Wuchang City Center for Tuberculosis Control and Prevention, Harbin, People’s Republic of China; h School of Public Health (Shenzhen), Shenzhen Campus of Sun Yat-sen University, Shenzhen, People’s Republic of China; i Laboratorio de Genética Molecular, CMBC, IVIC, Caracas, Venezuela

**Keywords:** Tuberculosis, transmission, social contacts, whole-genome sequencing, rural China

## Abstract

Tuberculosis (TB) is more prevalent in rural than urban areas in China, and delineating TB transmission patterns in rural populations could improve TB control. We conducted a prospective population-based study of culture-positive pulmonary TB patients diagnosed between July 1, 2009 and December 31, 2020 in two rural counties in China. Genomic clusters were defined with a threshold distance of 12-single-nucleotide-polymorphisms, based on whole-genome sequencing. Risk factors for clustering were identified by logistic regression. Transmission links were sought through epidemiological investigation of genomic-clustered patients. Of 1517 and 751 culture-positive pulmonary TB patients in Wusheng and Wuchang counties, respectively, 1289 and 699 strains were sequenced. Overall, 624 (31.4%, 624/1988) patients were grouped into 225 genomic clusters. Epidemiological links were confirmed in 41.8% (196/469) of clustered isolates, including family (32.7%, 64/196) and social contacts (67.3%, 132/196). Social contacts were generally with relatives, within the community or in shared aggregated settings outside the community, but the proportion of clustered contacts in each category differed between the two sites. The time interval between diagnosis of student cases and contacts was significantly shorter than family and social contacts, probably due to enhanced student contact screening. Transmission of multidrug-resistant (MDR) strains was likely responsible for 81.4% (83/102) of MDR-TB cases, with minimal acquisition of additional resistance mutations. A large proportion of TB transmission in rural China occurred among social contacts, suggesting that active screening and aggressive contact tracing could benefit TB control, but contact screening should be tailored to local patterns of social interactions.

## Introduction

Tuberculosis (TB) remains an important threat to global health, with an estimated 10 million people worldwide falling ill and at least 1.4 million deaths in 2019 [1]. Although the global incidence of TB has declined in recent years, there is still a significant gap between current rates of decline and the goals of the WHO End TB Strategy [2,3].

It is estimated that approximately one-third of TB patients worldwide were not diagnosed [1], so measures that increase diagnosis, especially early in the disease, are key to reducing transmission and thereby controlling TB. One of the core strategies to increase TB diagnosis and achieve global TB control is active case-finding [4]. The World Health Organization (WHO) recommends systematic TB screening of the general population in areas with an estimated TB prevalence of 0.5% or higher [5]. For areas with a lower TB prevalence, a cost-effective strategy involves identifying populations at high risk for transmission and then implementing targeted screening.

Genomic-epidemiological studies of tuberculosis can help identify populations at high risk of transmission, but the limited discriminatory power of previously employed genotyping methods and the barriers to epidemiological investigations have made it difficult to define these populations in China [6]. The populations at high risk for TB in other countries, such as HIV-positive, homeless, and drug abusing individuals, are less important contributors to the TB burden in China [5,7]. Instead, TB screening in China is focused principally on the elderly and internal migrants, but because of the large size of these populations in China, generalized screening is not practical. Most genomic-epidemiological studies of tuberculosis in China have been conducted in large cities, but China also has extensive rural areas where the prevalence of TB is up to three times higher than in urban regions [8]. Therefore, identifying high-risk populations and delineating transmission patterns in rural areas is important for improving TB control in China. To define the transmission patterns of the rural TB burden and thereby provide guidance for improving TB control, we conducted a 12-year prospective population-based genomic-epidemiological study in two rural counties in China.

## Methods

### Study design and participants

Wusheng and Wuchang are rural counties in southwestern and northeastern China, respectively (Supplementary Figure S1). Wusheng is located in the east of Sichuan Province, with an area of 960 square kilometres and an estimated 556,000 inhabitants in 2020. Wuchang is located in the south of Heilongjiang Province, with an area of 7,512 square kilometres. The study included 14 townships (2,299.5 square kilometres) in Wuchang, with a combined estimated 419,000 inhabitants in 2020. The average annual reported TB incidences were 95.8 (per 100,000 population) in Wusheng and 68.9 in Wuchang (Supplementary Figure S2). Lower incidences recorded in 2020 were probably inaccurate and a result of reduced TB diagnosis due to the COVID-19 pandemic.

The study population was comprised of all culture-positive pulmonary TB patients 15 years or older who were diagnosed between July 1, 2009 and December 31, 2020 in the regions. The study was approved by the institutional review board of Biomedical Sciences, Fudan University and all enrolled patients provided written informed consent.

### Diagnostic procedures

The Wusheng County Centre for Disease Control and Prevention and the Wuchang City Centre for Tuberculosis Control and Prevention are responsible for the diagnosis and treatment of TB in their respective areas. Individuals with TB-like symptoms or abnormal chest radiographs in general hospitals and township health centres are referred to these designated medical institutions for diagnosis by sputum smear and culture. Sputum specimens were collected when the patients presented for TB diagnosis, before starting treatment. They were cultured in Löwenstein-Jensen media during 2009–2016 and in liquid media during 2017-2020. Culture-positive isolates were inactivated at 80 °C for 30 min and stored in the −20 °C refrigerator of the site laboratory.

### Whole-genome sequencing

All stored clinical strains isolated during the study period were re-cultured from frozen stocks. DNA was extracted using the cetyl trimethyl ammonium bromide (CTAB) method and sequenced as previously described [9]. Raw sequence reads were trimmed with Sickle (version 1.33) and aligned to the inferred *Mycobacterium tuberculosis* complex ancestor sequence [10] using BWA-MEM. SAMtools (version 1.3.1) and Varscan (version 2.3.6) were then used to identify single nucleotide polymorphisms (SNPs). Pairwise SNP distances were calculated based on fixed SNPs (frequency ≥75%), excluding those in drug-resistance associated genes and repetitive regions of the genome (e.g. PPE/PE-PGRS family genes, phage sequences, and insertion or mobile genetic elements). A genomic cluster was defined as strains differing by 12 or fewer SNPs, consistent with linkage through recent transmission [11].

Based on the identified SNPs, a phylogeny tree was constructed with RAxML-NG (version 1.0.2) software, using the maximum likelihood method with 100 bootstraps and visualized with Interactive Tree of Life (https://itol.embl.de/). Strains were classified into different lineages according to Liu et al [12]. Strains belonging to lineage 2 (L2), also termed the Beijing family, were divided into L2.3, representing “modern” Beijing and other sub-lineages considered “ancient” Beijing [13].

Drug-resistance profiles were predicted for 14 anti-TB drugs based on the mutations reported to be associated with resistance [14], using Whole-genome sequencing (WGS) data as described previously [13]. Pan-susceptible TB was defined as strains without mutations associated with resistance to the four first-line drugs (isoniazid, rifampicin, ethambutol and pyrazinamide). Multidrug-resistant TB (MDR-TB) was defined as strains with mutations associated with resistance to at least isoniazid and rifampicin. Strains with mutations associated with resistance to any of the four first-line drugs but not MDR were termed other drug resistant (DR) TB.

### Epidemiological investigation

We conducted epidemiological investigations twice. First, at the time of TB diagnosis we collected demographic, clinical and laboratory information on each patient and also collected data on their close contacts. We then conducted in-depth epidemiological investigation of patients whose strains belonged to genomic clusters, including their residences, workplaces and public community centres and facilities frequented in the three years prior to their TB diagnosis. Putative transmission networks were constructed based on the structure of the genomic phylogeny and the epidemiologic links. The epidemiologic links were defined as confirmed – patients who knew each other and had a history of contact before diagnosis, or probable – patients who did not know each other but lived in the same village. We classified the confirmed epidemiological links into 4 categories according to the closeness of the relationship between the patients: family – the patients were immediate relatives; relatives – the patients were related but not immediate relatives; community – patients lived in the same village; and aggregated settings outside the community – patients who had contact outside the community in settings such as teahouses, psychiatric hospitals, schools and nursing homes.

### Statistical analysis

The clustering rate was calculated by the N method and the cumulative clustering rate was obtained by calculating the clustering rate in 2009 and then adding the TB patients from every successive year of the study, as previously described [15]. Changes in temporal trends of annual clustering were detected using joinpoint regression analysis (Supplementary methods). The distribution of continuous and categorical variables between groups was compared using the Wilcoxon rank sum test or the chi-square test. Logistic regression analysis was used to calculate the odds ratios (OR) and 95% confidence intervals (CI) for risk factors associated with genomic clustering. Variables with *p*-values less than 0.2 in the univariable analysis were included in the multivariable analysis to calculate the adjusted odds ratios (aOR). Factors with a *p*-value less than 0.05 in the final model were considered statistically significant. All analyses were performed in Stata (version 14.0).

## Results

### General population characteristics

**Figure 1.**
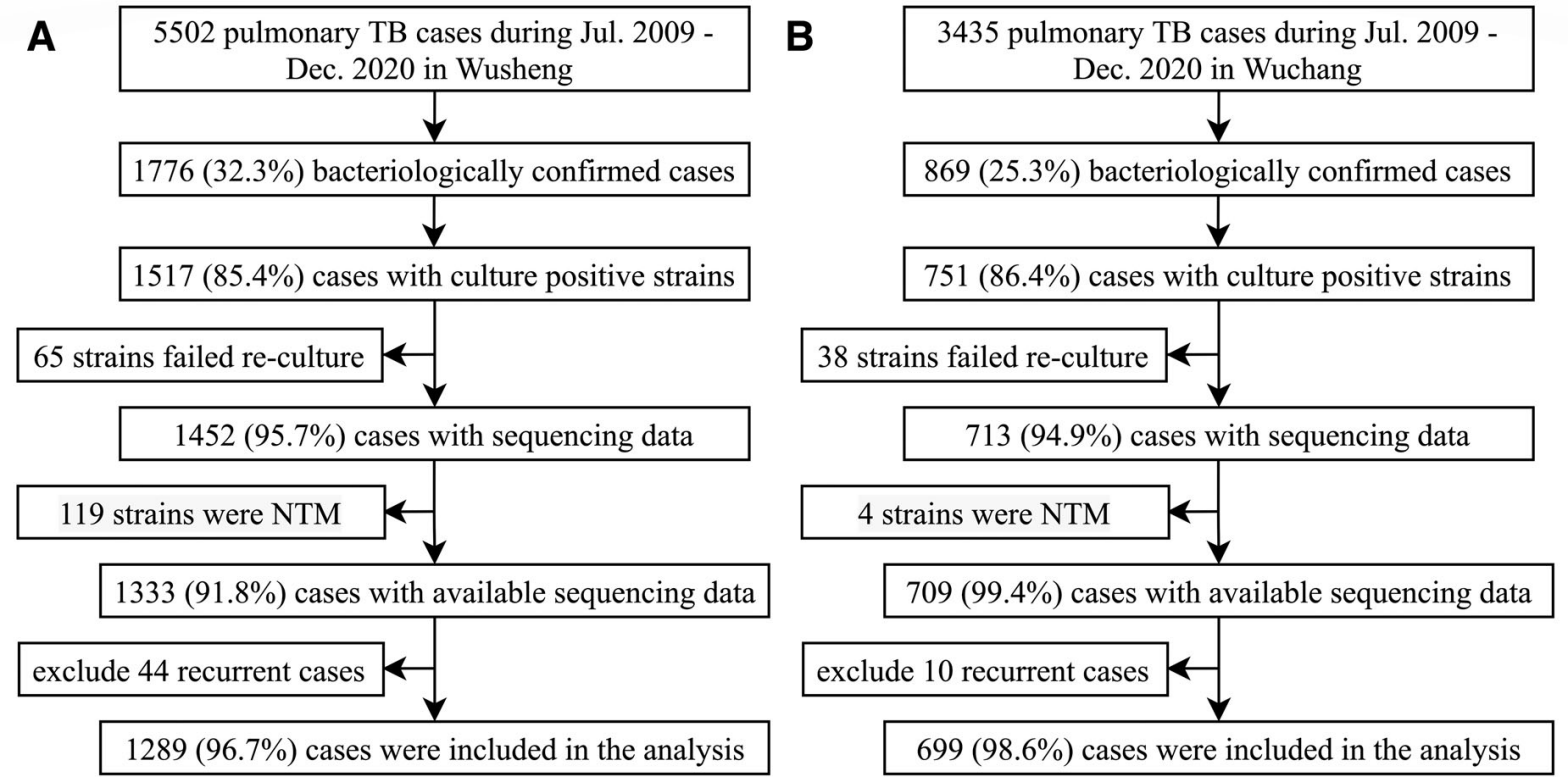
Sample enrolment in (A) Wusheng and (B) Wuchang. NTM: non-tuberculous mycobacteria.

Between July 1, 2009 and December 31, 2020, 5502 and 3435 pulmonary TB patients were officially registered in Wusheng and Wuchang, respectively, of whom 1517 and 751 had positive sputum cultures. After excluding 103 strains that failed re-culturing, 1452 and 713 strains were sequenced. We further excluded 123 patients with non-tuberculous mycobacteria isolates and 54 patients with recurrent TB. The final analysis thus included 1289 and 699 patients from Wusheng and Wuchang, respectively (Figure 1), whose characteristics are shown in Table 1. The proportions of patients who were students or less than 25 years old were higher in Wusheng [15.4% (198/1289) and 7.6% (98/1289)] than in Wuchang [9.9% (69/699) and 2.9% (20/699)]. Patients in Wuchang were more likely to have had longer diagnosis delays, chest cavities and smear positivity.

**Table 1.** Characteristics of tuberculosis patients in Wusheng and Wuchang, 2009-2020.

Characteristics	Wusheng (%)	Wuchang (%)
Total	1289	699
Sex		
Female	271 (21.0)	202 (28.9)
Male	1018 (79.0)	497 (71.1)
Age		
<25	198 (15.4)	69 (9.9)
25–44	328 (25.4)	193 (27.6)
45–64	519 (40.3)	296 (42.3)
≥65	244 (18.9)	141 (20.2)
Occupation		
Farmer	1096 (85.0)	578 (82.7)
Students	98 (7.6)	20 (2.9)
Others	95 (7.4)	101 (14.4)
History of tuberculosis		
New	1197 (92.9)	642 (91.8)
Retreated	92 (7.1)	57 (8.2)
Diagnostic delay		
<2 weeks	428 (33.2)	103 (14.7)
2–4 weeks	227 (17.6)	162 (23.2)
4–8 weeks	371 (28.8)	208 (29.8)
≥8 weeks	263 (20.4)	226 (32.3)
Chest cavitation		
No	889 (69.0)	407 (58.2)
Yes	400 (31.0)	292 (41.8)
Sputum smear status		
Negative	634 (49.2)	244 (34.9)
Positive	655 (50.8)	455 (65.1)
Drug-resistance profile		
Pan-susceptible	1128 (87.5)	588 (84.1)
Other DR	95 (7.4)	75 (10.7)
MDR	66 (5.1)	36 (5.2)

DR: drug resistance; MDR: multidrug resistance.

### Whole genome sequencing for genotyping and drug resistance prediction

**Figure 2.**
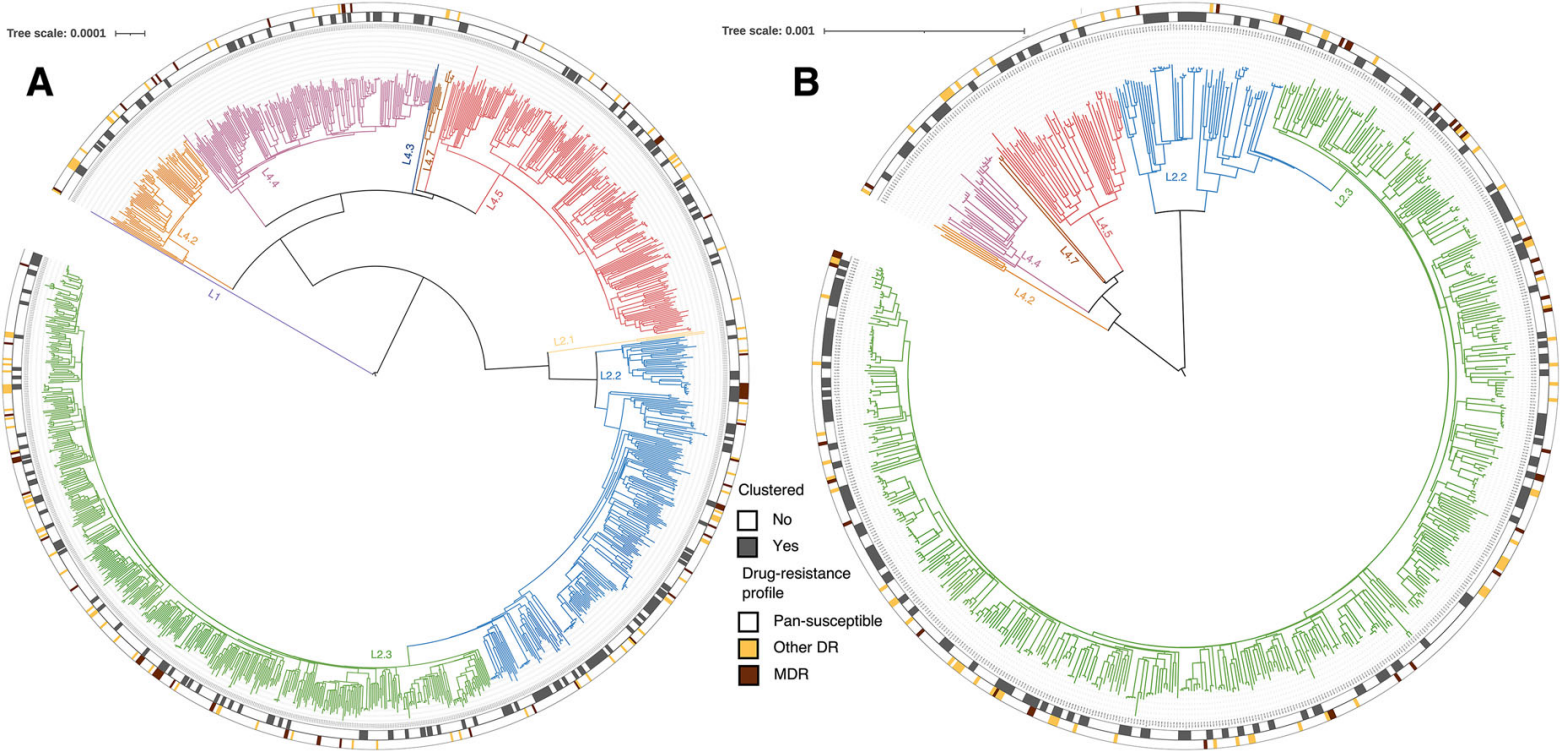
Phylogeny, clustering, and resistance profile of 1289 *Mycobacterium tuberculosis* strains isolated in (A) Wusheng and 699 *Mycobacterium tuberculosis* strains isolated in (B) Wuchang. The different colours on the branches indicate different lineages and sublineages. The outer grey circle indicates genomic-clustered strains differing by ≤ 12 single-nucleotide polymorphisms. The outer yellow-red circle indicates other drug resistance and multidrug resistance.

Phylogenetic analysis revealed that most strains belonged to the Beijing family lineage (69.0%, 1372/1988) (Figure 2), with more belonging to the modern Beijing sublineage (74.1%, 1016/1372) than ancient Beijing sublineages (25.9%, 356/1372). All of the non-Beijing strains belonged to L4 (99.8%, 615/616), except for one L1 strain. Analysis of the genome sequences for mutations conferring resistance to 14 anti-TB drugs showed that the drug-resistance profiles were similar between the two sites (Figure 2; Table 2; Supplementary Table S1). In total, there were 1716 (86.3%) pan-susceptible strains and 272 (13.7%) strains with mutations associated with resistance to at least one anti-TB drug. The MDR strains accounted for 5.1% (102/1988) of the total strains, and 29.4% (30/102) of MDR strains were predicted to harbour mutations associated with resistance to the fluoroquinolones.

**Table 2.** Drug-resistance profile, stratified by new and retreated cases.

	New cases (%)	Retreated cases (%)	Total (%)
Total	1839	149	1988
Pan-susceptible	1610 (87.5)	106 (71.1)	1716 (86.3)
With mutations associated with resistance to INH	180 (9.8)	32 (21.5)	212 (10.7)
With mutations associated with resistance to RIF	119 (6.5)	33 (22.1)	152 (7.6)
With mutations associated with resistance to EMB	54 (2.9)	22 (14.8)	76 (3.8)
With mutations associated with resistance to PZA	24 (1.3)	9 (6.0)	33 (1.7)
MDR	78 (4.2)	24 (16.1)	102 (5.1)
MDR and with mutations associated with resistance to FQ	24 (1.3)	6 (4.0)	30 (1.5)

INH: isoniazid; RIF: rifampicin; EMB: ethambutol; PZA: pyrazinamide; MDR: multidrug resistance; FQ: fluoroquinolone.

### Genomic clustering analysis of *M. tuberculosis*

**Figure 3.**
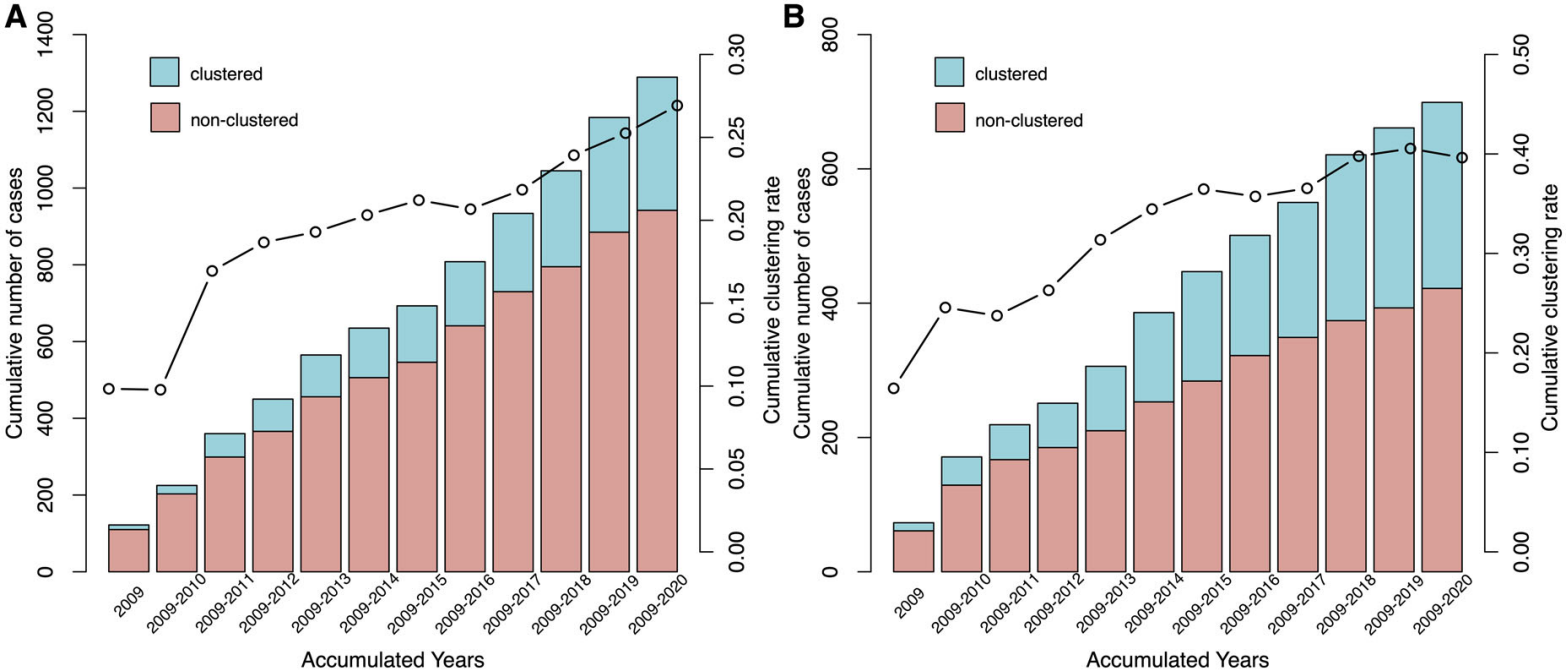
Cumulative clustering rate in (A) Wusheng and (B) Wuchang, 2009-2020. The bar indicates the cumulative number of cases, the line indicates the cumulative clustering rate.

To estimate the level of recent transmission, we calculated the clustering rate between 2009–2020. A total of 624 (31.4%, 624/1988) strains, 347 (26.9%, 347/1289) from Wusheng and 277 (39.6%, 277/699) from Wuchang, were grouped into 225 genomic clusters containing 2–13 strains (Figure 2). To understand the dynamics of clustering trends in the two sites, we calculated the cumulative clustering rate and found that the rate gradually increased as more strains were analyzed, but the trend eased between 2015 and 2016 in both sites. As seen in Figure 3, the clustering rate increased significantly after 2016, especially in Wusheng (*p* = 0.004, Supplementary Figure S3), rising from 21.8% in 2017–26.9% in 2020. The increased clustering rate may have been the result of strategies we implemented starting in 2017: active case-finding based on symptoms that identified 15-25% more patients; stepwise sputum collection that improved the diagnostic quality of the sputum [16]; and sputum cultures in liquid media that increased the percentage of culture-positive patients from 20-30% to 40-50% (Supplementary Figure S4).

### Analysis of MDR-TB

In the analysis of risk factors for MDR-TB, the only association was a higher proportion of MDR in retreated patients (16.1%, 24/149) than in new cases (4.2%, 78/1839) (Supplementary Table S2). To investigate the cause of MDR we analyzed the number of clustered and non-clustered patients in the 102 MDR patients and found that 34 were clustered. While retreated MDR-TB cases could have secondary resistance that was acquired during treatment, new cases were presumably infected with MDR strains and represent primary resistance. Among the non-clustered MDR patients, 49 were new patients. Therefore, in total, 81.4% (83/102) of MDR-TB patients were likely caused by the transmission of MDR strains. We also looked for accumulated additional drug-resistance mutations along the transmission chain. Among 32 putative MDR-TB transmission in 11 genomic-clusters, we found just two events (6.3%, 2/32) indicating the acquisition of additional resistance (Supplementary Figure S5), which is lower than the incidence of acquired resistance reported in urban areas of China [11].

### Epidemiological links of genomic-clustered patients

To further delineate the transmission links between the genomic-clustered patients we performed an in-depth epidemiological investigation on all patients with clustered isolates. These investigations were completed for 469 (75.2%, 469/624) patients, 294 in Wusheng and 175 in Wuchang, but the other 155 clustered patients (24.8%) had either died or were lost to follow-up. Confirmed epidemiological links were identified in 196 (41.8%, 196/469) of the genomic-clustered patients investigated (110 in Wusheng and 86 in Wuchang), and probable epidemiological links were identified in 26 (5.5%, 26/469) patients. The characteristics of each cluster with confirmed or probable epidemiological links are shown in Supplementary Tables S3 and S4.

**Figure 4.**
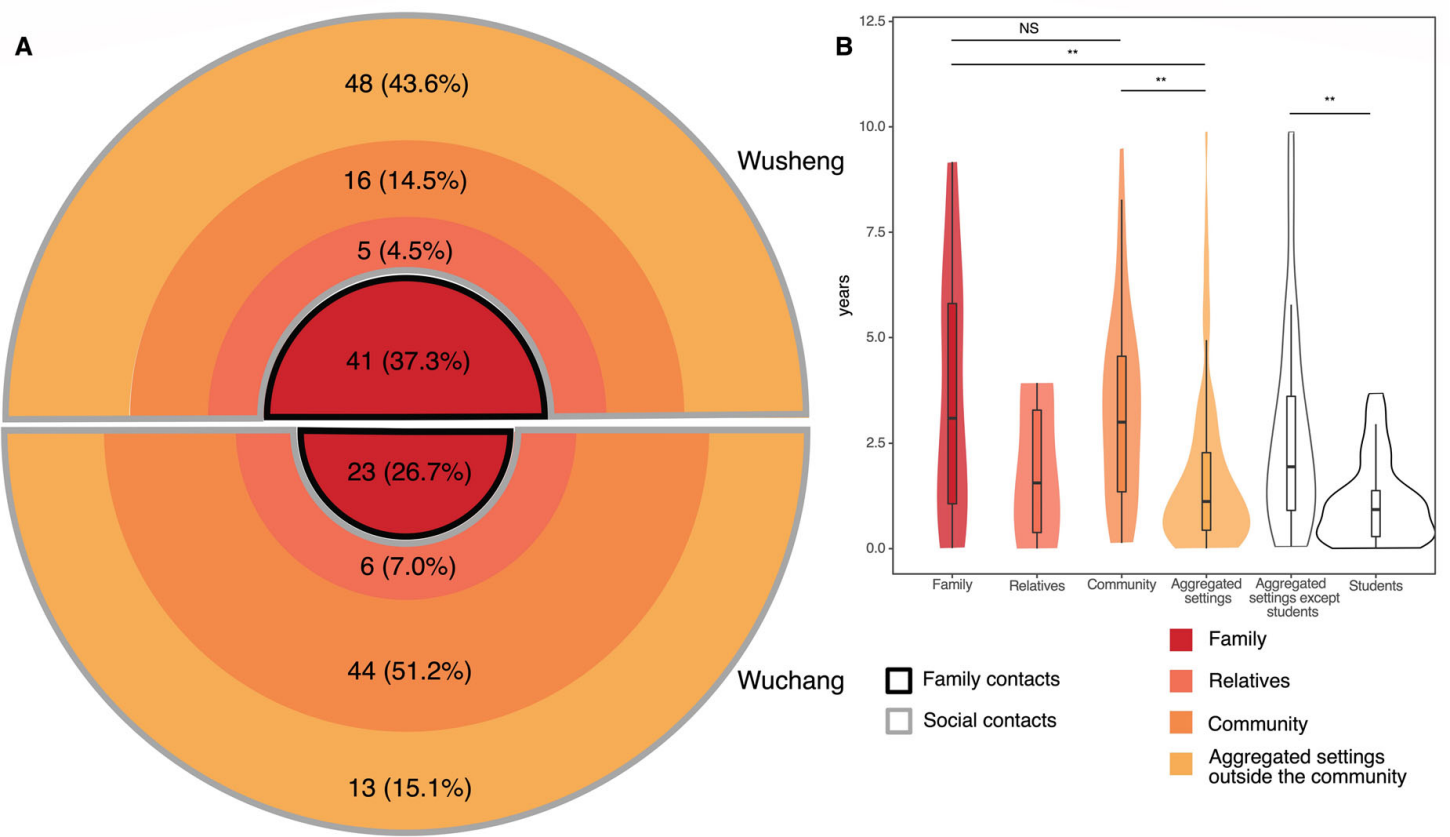
Categories of confirmed epidemiological links and their time interval between diagnoses. The four colours of the circle indicate four categories of contacts with confirmed epidemiological links. The darker the colour, the closer the relationship. (A) The black border indicates family contacts (Family) and the grey border indicates social contacts (Relatives, Community and Aggregated settings outside the community). The number in the ring indicates the number and proportion of each category of contacts. The width of the ring indicates the proportion of each category. (B) The time interval between diagnoses in the four categories of contacts and students. Due to the small number of relatives, they were not compared with the other categories. **p < 0.01, NS refers to no significance (given by Wilcoxon rank sum test).

The 196 patients with confirmed epidemiological links were all close contacts. Among them (Figure 4A), transmission between family contacts was identified in 32.7% (64/196) and between social contacts in 67.3% (132/196). To further analyze transmission patterns, we divided the social contacts into three categories: relatives outside of the immediate family, community contacts, and contacts who shared aggregated settings such as teahouses located outside the community. Epidemiological links were found in community contacts in 14.5% (16/110) of the confirmed transmission in Wusheng but 51.2% (44/86) in Wuchang. Surprisingly, the proportions were reversed for confirmed epidemiological links in aggregated settings outside the community, with 43.6% (48/110) in Wusheng and 15.1% (13/86) in Wuchang. The percentages of confirmed links to relatives beyond the immediate family were low in both sites, with 4.5% (5/110) in Wusheng and 7.0% (6/86) in Wuchang.

**Figure 5.**
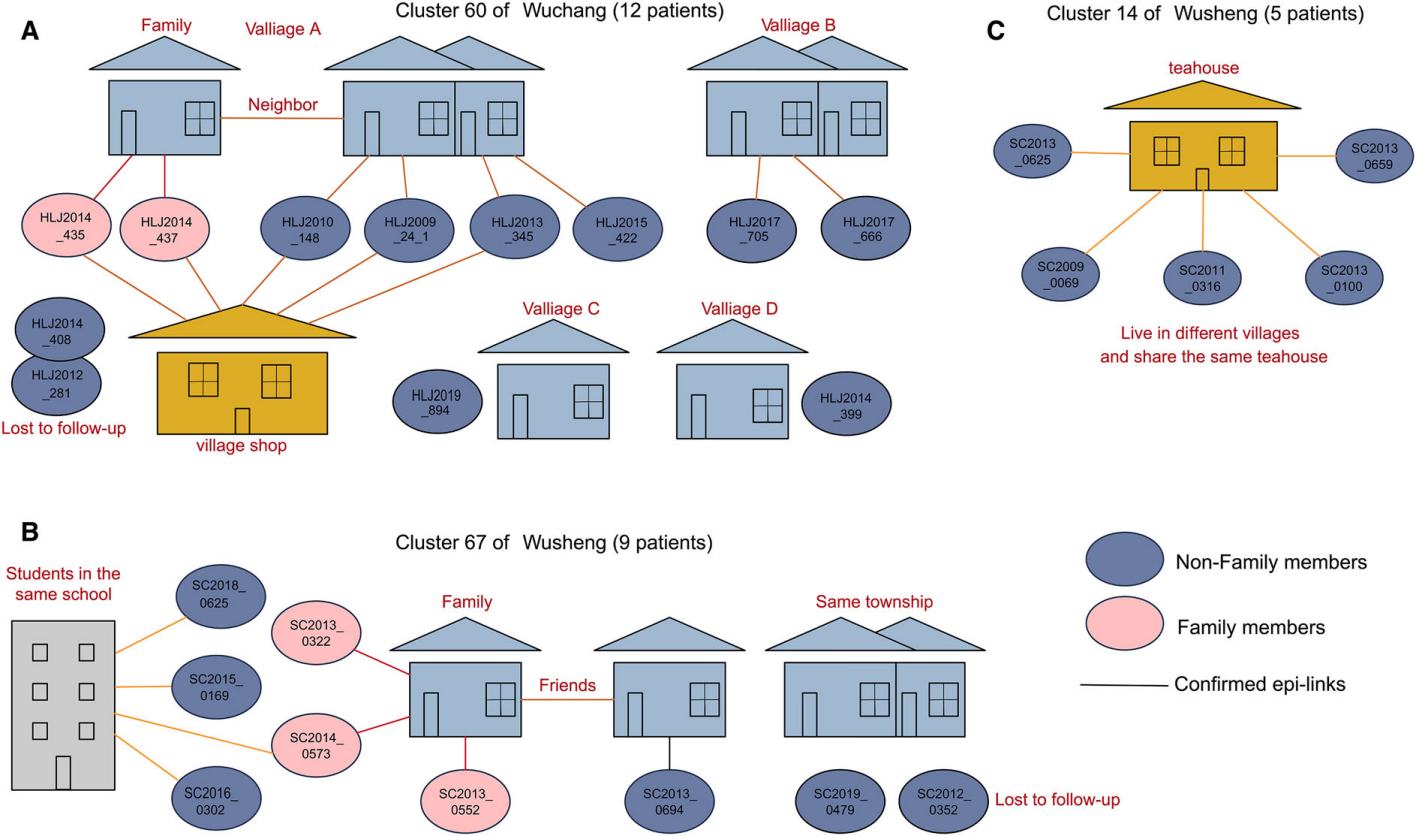
Putative transmission network for 3 clusters based on the structure of the genomic phylogeny and the epidemiological links. (A) Transmission network of Cluster 60 of Wuchang. HLJ2014_435, HLJ2014_437, HLJ2010_148, HLJ2009_24_1, HLJ2013_345 and HLJ2015_422 all live in village A. HLJ2014_435 and HLJ2014_437 are couples and they often go to the village shop to chat and play card. HLJ2010_148, HLJ2009_24_1, HLJ2013_345 and HLJ2015_422 are neighbours of HLJ2014_435 and HLJ2014_437. HLJ2010_148, HLJ2009_24_1 and HLJ2013_345 also often go to the village shop to chat and play card. HLJ2017_705 and HLJ2017_666 live in village B and know each other. HLJ2019_894 and HLJ2014_399 live in village C and village D, and no epidemiological links was found with other patients. (B) Transmission network of Cluster 67 of Wuchang. SC2013_0552 and SC2013_0694 are friends. SC2013_0322 and SC2014_0573 are grandsons of SC2013_0552. SC2015_0169, SC2016_0302 and SC2018_0169 are classmates of SC2014_0573. (C) Transmission network of Cluster 14 of Wuchang. SC2013_0625, SC2009_0069, SC2011_0316, SC2013_0100 and SC2013_0659 live in different villages. They know each other and go to the teahouse in the township almost every day to drink tea, chat and play mahjong. The colour of the line indicates the categories of contacts in Figure 4. Epi-link: epidemiological link.

We constructed putative transmission networks based on genomic phylogeny and epidemiological links. Figure 5 shows examples of three of the largest clusters. The inhabitants of the villages in Wuchang live in fairly close proximity, often with daily contact in village shops that may explain the high level of transmission occurring within the community (Figure 5A). In Wusheng, by contrast, the majority of identified epidemiological links were outside the community and mainly in schools and teahouses (Figure 5B and C). The teahouses in Wusheng are generally located in townships outside the villages and are frequented by people from several different villages.

### Time interval between TB diagnosis

We then analyzed the pairwise time interval between TB diagnosis in the clustered cases belonging to the different categories of contacts (Figure 4B). We found no statistical difference (*p* = 0.90) in the time interval between diagnosis of family contacts (3.1 years, interquartile range [IQR] 0.9-5.9 years) and community contacts (3.0 years, IQR 1.3-4.6 years). Unexpectedly though, the average diagnostic time interval between contacts who shared the aggregated settings outside the community (1.1 years, IQR 0.4-2.3 years) was significantly shorter than for family (*p* < 0.01) or community contacts (*p* < 0.01). This shorter interval was perhaps because most of the contacts who shared aggregated settings outside the community were students, and the average time interval between the diagnosis of student contacts (0.9 years, IQR 0.3-1.4 years) was significantly shorter (*p* < 0.01) than for all other contacts (1.9 years, IQR 0.7-3.6 years).

### Risk factors for genomic clustering

Finally, we used logistic regression to identify risk factors associated with clustering. The univariate analysis found that occupation and the Beijing strain were significantly associated with clustering, and both associations persisted in the multivariate analysis (Table 3). Patients with a Beijing lineage strain had a greater risk of clustering (aOR 1.46, 95% CI, 1.17-1.82, *p* *= *0.001) than patients with L4 strains. Also, compared to any other occupation, farmers had a lower risk (aOR 0.60, 95% CI, 0.44-0.82, *p* = 0.001) and students had a higher risk (aOR, 2.11; 95% CI, 1.20-3.71, *p* = 0.01) of clustering.

**Table 3.** Univariate and multivariable logistic regression of risk factors for clustering.

	Clustered (%)	Non-clustered (%)	Univariate regression		Multivariable regression	
			OR (95% CI)	*p* value	aOR (95% CI)	*p* value
Total	624	1364				
Sex						
Female	148 (31.3)	325 (68.7)	1.00		..	
Male	476 (31.4)	746 (68.6)	1.01 (0.81, 1.26)	0.958	..	..
Age						
<25	117 (43.8)	150 (56.2)	2.41 (1.73, 3.38)	<0.001	1.35 (0.89, 2.06)	0.160
25–44	164 (31.5)	357 (68.5)	1.42 (1.06, 1.91)	0.020	1.35 (1.00, 1.82)	0.054
45–64	249 (30.6)	566 (69.4)	1.36 (1.03, 1.80)	0.028	1.34 (1.01, 1.77)	0.041
≥65	94 (24.4)	291 (75.6)	1.00		1.00	
Occupation						
Farmer	475 (28.4)	1199 (71.6)	0.56 (0.42, 0.76)	<0.001	0.60 (0.44, 0.82)	0.001
Students	68 (57.6)	50 (42.4)	1.93 (1.22, 3.07)	0.005	2.11 (1.20, 3.71)	0.010
Others	81 (41.3)	115 (58.7)	1.00		1.00	
History of tuberculosis						
New	582 (31.6)	1257 (68.4)	1.00		..	
Retreated	42 (28.2)	107 (71.8)	0.85 (0.59, 1.23)	0.382	..	..
Diagnostic delay						
<2 weeks	157 (29.6)	374 (70.4)	1.00		..	
2–4 weeks	124 (31.9)	265 (68.1)	1.11 (0.84, 1.48)	0.452	..	..
4–8 weeks	188 (32.5)	391 (67.5)	1.15 (0.89, 1.48)	0.297	..	..
≥8 weeks	155 (31.7)	334 (68.3)	1.11 (0.85, 1.44)	0.461	..	..
Chest cavitation						
No	385 (29.7)	911 (70.3)	1.00		1.00	
Yes	239 (34.5)	453 (65.5)	1.25 (1.03, 1.52)	0.027	1.22 (1.00, 1.50)	0.054
Sputum smear status						
Negative	258 (29.4)	620 (70.6)	1.00		1.00	
Positive	366 (33.0)	744 (67.0)	1.18 (0.98, 1.43)	0.087	1.19 (0.98, 1.46)	0.085
Drug-resistance profile						
Pan-susceptible	547 (31.9)	1169 (68.1)	1.00		1.00	
Other DR	43 (25.3)	127 (74.7)	0.72 (0.50, 1.04)	0.079	0.68 (0.47, 0.98)	0.040
MDR	34 (33.3)	68 (66.7)	1.07 (0.70, 1.63)	0.759	1.00 (0.65, 1.54)	1.000
Beijing strain						
No	158 (25.6)	458 (74.4)	1.00		1.00	
Yes	466 (34.0)	906 (66.0)	1.49 (1.21, 1.84)	<0.001	1.46 (1.17, 1.82)	0.001

OR: odds ratio; aOR: adjusted odds ratio; DR: drug resistance; MDR: multidrug resistance.

## Discussion

To our knowledge, this was the longest longitudinal population-based genomic epidemiological study of TB transmission in rural China. The overall cumulative clustering rate during the study period was 31.4%. Among 196 genomic-clustered patients with confirmed epidemiological links, 32.7% of transmission occurred between family contacts and 67.3% between social contacts. Of all MDR-TB cases diagnosed during the study, 81.4% were likely from transmission of MDR strains. The average time interval between the diagnosis of clustered student contacts was much shorter than for non-student contacts.

Close contacts of TB patients are at a high-risk for TB transmission. A recent systematic review suggested that the pooled prevalence of TB in close contacts was 3.6% (95% CI: 3.3-4.0), and that contact investigation could increase TB case notification and thereby decrease the incidence of TB in the population [17]. In developed countries, index cases have been found to have 6–12 close contacts [18–21] who could potentially constitute 10-20% [22,23] of the total TB burden. Accordingly, the WHO strongly recommends systematic screening for TB disease among close contacts [5,24]. In China, however, the importance of screening close contacts has been underappreciated and its role in case finding has been limited. In China, only 2–3 close contacts per index case were identified and most of these were family members. It was therefore estimated that close contacts would contribute only about 1.0-3.3% of the total TB patients in China [25,26]. A recent community-based study of active case-finding that included 320,000 people in 10 provinces in China during 2013–2015 concluded that screening of close contacts contributed less then 2% of TB patients [7].

Our study found that 41.8% of clustered patients were close contacts of other patients and contributed 9.9% of the total TB patients, suggesting a high risk of TB transmission among close contacts in rural China. However, it was only through in-depth investigation of these genomic-clustered patients that we could identify the epidemiological links that are usually overlooked in routine contact investigation. In contrast to our findings, the routine TB contact investigations that were conducted in the study sites during the duration of our study registered only 2.4 close contacts per index case, who contributed just 1.8% of the total patients. Without the WGS data that identified clustered patients and directed our investigations, nearly 80% of the transmission events between close contacts would have been missed. Most secondary cases were diagnosed between 1–3 years after the index case, but the long study duration of 12 years, using WGS to analyze all TB isolates in the study areas, allowed us to identify clustered isolates that would be missed in studies with a shorter duration.

Screening of close contacts in China has been poorly implemented for a number of reasons. The huge number of TB patients, limited resources for TB prevention and control and the stigma of tuberculosis all contribute to a failure to identify and screen many close contacts. When contact screening is poorly implemented, the contribution of close contacts to the overall TB burden is underestimated and therefore less attention is paid to the screening, creating a vicious cycle. WHO guidelines define close contacts as persons sharing an enclosed space with the index case during the three months prior to commencement of the current treatment episode [24]. While the identification of family contacts is generally straightforward, the definition and identification of non-family contacts is difficult. In China, non-family contacts refer mainly to classmates and colleagues [26]. In this study, however, we found that many patients with confirmed epidemiologic links were contacts either within the community or contacts who shared aggregated settings outside the community. Therefore, in addition to family contacts, we classified others as social contacts in the hope of understanding where contacts occur and highlighting their relevance for TB transmission in rural China.

We found that in rural China 67.3% of the transmission occurred among social contacts, which was significantly higher than has been found in Vietnam, Zambia and South Africa (15-50%) [27,28]. This suggests that active case-finding in rural China should go beyond family contacts to include social contacts. In this study we proposed three categories of social contacts: relatives outside of the immediate family; people who know each other and live in the same community or village; and people frequenting aggregated settings outside the community. Largely due to differences in climate and lifestyle, the importance of the different categories of contacts varied between the two study sites (Supplementary Figure S6). The villages in Wuchang are very small, consisting of perhaps only a dozen families living in houses that are generally adjacent to each other. Wuchang is located in the northeast of China and has an average annual temperature of only 4°C. Consequently, villagers spend more than half of the year indoors and frequently interact to socialize and play cards with other villagers in the village shops. It is therefore not surprising that 51.2% of epidemiological links were attributable to transmission between social contacts within these communities. Wusheng, in contrast, is located in the southwest of China with an average annual temperature of 18°C. and villagers live in homes that are relatively scattered in the region. We found that transmission between villagers in this setting was less common than transmission at aggregated settings such as schools and teahouses, where individuals from several different villages congregate. These differences in transmission between the two locations suggests that strategies for screening and social contact tracing must be tailored to the conditions and association habits of the local populations.

The purpose of active case-finding is to diagnose patients early and reduce transmission. Importantly, we found that the time interval between TB diagnosis of student patients and contacts was 3–4 times shorter than for family or community contacts (Figure 4B). Students are a demographic group in China that receives special attention from the government and society. Once a student patient is diagnosed with TB, their classmates and even schoolmates will be screened. If a similar enhanced screening strategy could be extended to other social contacts, patients might be diagnosed earlier in the course of their disease, thereby reducing rural TB transmission.

This study have several limitations. The most important limitation is that the clustering rate we calculated likely underestimates the extent of local TB transmission. We previously found that the current passive case-finding strategy in Wusheng identified only about 30% of the incident TB patients [29], but in the current study we were unable to even estimate the number of TB patients in the sampling region who were undiagnosed or diagnosed elsewhere. Some of the non-clustered patients could have been clustered with TB patients diagnosed locally but without cultured isolates, or with patients diagnosed outside the study period or the geographic regions sampled. In addition, some clustered patients died or were lost to follow-up before the epidemiological investigations could be completed.

In conclusion, this long-term genomic-epidemiological study helped to delineate the patterns of TB transmission in rural China. Transmission appears to occur principally among close contacts, and therefore contact investigation should be extended to include the social interactions that are common in the targeted population. Further improvement in contact tracing, especially the identification and screening of all close contacts, is critical for reducing transmission and improving TB control in rural areas.

## Supplementary Material

Supplemental Material

## Data Availability

Sequencing data were deposited in the Genome Sequence Archive (https://bigd.big.ac.cn/gsa) under BioProject PRJCA008815 and PRJCA008816. De-identified participant data from the study will be made available with publication to medical researchers on a not for profit basis by email request to the corresponding author for the purposes of propensity matching or meta-analysis.

## References

[1] World Health Organization. Global tuberculosis report 2020. Geneva: World Health Organization; 2020.

[2] Churchyard G, Kim P, Shah NS, et al. What We know about tuberculosis transmission: An overview. J Infect Dis 2017;216(suppl_6):S629–S635. 29112747 10.1093/infdis/jix362 PMC5791742

[3] Floyd K, Glaziou P, Zumla A, et al. The global tuberculosis epidemic and progress in care, prevention, and research: an overview in year 3 of the End TB era. Lancet Respir Med. 2018;6(4):299–314. 29595511 10.1016/S2213-2600(18)30057-2

[4] World Health Organization. The end TB strategy. Geneva: World Health Organization; 2014.

[5] World Health Organization. WHO consolidated guidelines on tuberculosis. module 2: screening – systematic screening for tuberculosis disease. Geneva: World Health Organization; 2021. 33822560

[6] Yang C, Gao Q. Recent transmission of mycobacterium tuberculosis in China: the implication of molecular epidemiology for tuberculosis control. Front Med. 2018;12(1):76–83. 29357036 10.1007/s11684-017-0609-5

[7] Zhang H, Cheng J, Yu YL, et al. Evaluation of the efectivenes of community-based pulmonary tuberculosis active case-finding among key populations: a multicenter prospective cohort study. Chin J Antituberc. 2021;43(12):1248–1259.

[8] Wang L, Zhang H, Ruan Y, et al. Tuberculosis prevalence in China, 1990-2010; a longitudinal analysis of national survey data. Lancet. 2014;383(9934):2057–2064. 24650955 10.1016/S0140-6736(13)62639-2

[9] Jiang Q, Liu Q, Ji L, et al. Citywide transmission of multidrug-resistant tuberculosis under China's rapid urbanization: A retrospective population-based genomic spatial epidemiological study. Clin Infect Dis 2020;71(1):142–151. 31504306 10.1093/cid/ciz790 PMC8127054

[10] Comas I, Coscolla M, Luo T, et al. Out-of-Africa migration and neolithic coexpansion of mycobacterium tuberculosis with modern humans. Nat Genet 2013;45(10):1176–1182. 23995134 10.1038/ng.2744 PMC3800747

[11] Yang CG, Luo T, Shen X, et al. Transmission of multidrug-resistant mycobacterium tuberculosis in Shanghai, China: a retrospective observational study using whole-genome sequencing and epidemiological investigation. Lancet Infect Dis. 2017;17(3):275–284. 27919643 10.1016/S1473-3099(16)30418-2 PMC5330813

[12] Liu Q, Ma A, Wei L, et al. China's tuberculosis epidemic stems from historical expansion of four strains of mycobacterium tuberculosis. Nat Ecol Evol. 2018;2(12):1982–1992. 30397300 10.1038/s41559-018-0680-6 PMC6295914

[13] Yang T, Wang Y, Liu Q, et al. A population-based genomic epidemiological study of the source of tuberculosis infections in an emerging city: shenzhen, China. Lancet Reg Health-W. 2021 Mar;8:100106. 10.1016/j.lanwpc.2021.100106 PMC8315418 34327429

[14] Papaventsis D, Casali N, Kontsevaya I, et al. Whole genome sequencing of mycobacterium tuberculosis for detection of drug resistance: a systematic review. Clin Microbiol Infect. 2017;23(2):61–68. 27665704 10.1016/j.cmi.2016.09.008

[15] Izumi K, Murase Y, Uchimura K, et al. Transmission of tuberculosis and predictors of large clusters within three years in an urban setting in Tokyo, Japan: a population-based molecular epidemiological study. BMJ Open. 2019 May 9;9(5):e029295. 10.1136/bmjopen-2019-029295 PMC6527980 31076478

[16] Jiang Q, Ji L, Qiu Y, et al. A randomised controlled trial of stepwise sputum collection to increase yields of confirmed tuberculosis. Int J Tuberc Lung Dis. 2019;23(6):685–691. 31315700 10.5588/ijtld.18.0524

[17] Velen K, Shingde RV, Ho J, et al. The effectiveness of contact investigation among contacts of tuberculosis patients: a systematic review and meta-analysis. Eur Respir J. 2021 Dec;58(6. 10.1183/13993003.00266-2021 34016621

[18] Denholm JT, Leslie DE, Jenkin GA, et al. Long-term follow-up of contacts exposed to multidrug-resistant tuberculosis in Victoria, Australia, 1995-2010. Int J Tuberc Lung Dis. 2012;16(10):1320–1325. 22863690 10.5588/ijtld.12.0092

[19] Hirsch-Moverman Y, Cronin WA, Chen B, et al. HIV counseling and testing in tuberculosis contact investigations in the United States and Canada. Int J Tuberc Lung Dis. 2015;19(8):943–953. 26162361 10.5588/ijtld.14.0642

[20] Young KH, Ehman M, Reves R, et al. Tuberculosis Contact Investigations–United States, 2003-2012. MMWR Morb Mortal Wkly Rep. 2016 Jan 1;64(50-51):1369–1374. 26720627 10.15585/mmwr.mm6450a1

[21] Izumi K, Ohkado A, Uchimura K, et al. Evaluation of tuberculosis contact investigations in Japan. Int J Tuberc Lung Dis. 2017;21(2):188–195. 28234083 10.5588/ijtld.16.0508

[22] Reichler MR, Khan A, Sterling TR, et al. Risk and timing of tuberculosis Among close contacts of persons with infectious tuberculosis. J Infect Dis 2018;218(6):1000–1008. 29767733 10.1093/infdis/jiy265 PMC6534268

[23] Martin-Sanchez M, Brugueras S, de Andres A, et al. Tuberculosis incidence among infected contacts detected through contact tracing of smear-positive patients. PLoS One. 2019;14(4):e0215322. 30986227 10.1371/journal.pone.0215322 PMC6464217

[24] World Health Organization. Recommendations for investigating contacts of persons with infectious tuberculosis in low- and middle-income countries. Geneva: World Health Organization; 2012. 24404639

[25] Gao CN, Tan QY, Xu ZW, et al. Analysis of TB case detection through investigation of close contacts of smear positive pulmonary tuberculosis patients. Chin J Antituberc. 2011;33(06):328–330.

[26] Jiang Q, Lu L, Wu J, et al. Assessment of tuberculosis contact investigation in Shanghai, China: An 8-year cohort study. Tuberculosis. 2018;108:10–15. 29523308 10.1016/j.tube.2017.10.001

[27] Horby P, Pham QT, Hens N, et al. Social contact patterns in Vietnam and implications for the control of infectious diseases. PLoS One. 2011;6(2):e16965. 21347264 10.1371/journal.pone.0016965 PMC3038933

[28] Dodd PJ, Looker C, Plumb ID, et al. Age- and Sex-specific social contact patterns and incidence of mycobacterium tuberculosis infection. Am J Epidemiol. 2016 Jan 15;183(2):156–166. 26646292 10.1093/aje/kwv160 PMC4706676

[29] Liao PJ, Qiu Y, Guo MC, et al. Analysis of patients from active case finding in rural areas of wusheng, sichuan. Chin J Antituberc. 2016;38(7):576–581.

