# Supplementary material for: High proportion of tuberculosis transmission among social contacts in rural China: a 12-year prospective population-based genomic epidemiological study
Source: Emerg Microbes Infect. 2022 Aug 31;11(1):2102–11. doi: 10.1080/22221751.2022.2112912 (PMC9448380; doi:10.1080/22221751.2022.2112912)
Supplement: Supplemental Material [file TEMI_A_2112912_SM6158.docx]

**Supplementary methods**

**Joinpoint regression analysis**

We used joinpoint regression analysis to detect the changes in temporal trends in cumulative clustering rate in Wusheng and Wuchang, and to estimate the annual percentage change (APC) and corresponding 95% CI for each trend segment.^1^ We also estimated the average annual percentage change (AAPC) assuming only one homogeneous trend over the entire range of our study period. This analysis was performed using Joinpoint software (version 4.9.0.1) developed by the Surveillance Research Program of the US National Cancer Institute (Bethesda, MD, USA).

**Results**

Supplementary Figure S3 presents the results of joinpoint regression analysis for cumulative clustering rate in Wusheng (left) and Wuchang (right). Cumulative clustering rate in Wusheng during the study period showed a persistent increase, with an AAPC of 11.5% (95% CI: 7.4-15.8%, P<0.001) between 2009 and 2020, presenting with three periods, including an APC of 47.2% (95% CI: 10.8-95.6%, P=0.010) between 2009 and 2011, an APC of 3.8 % (95% CI: 1.6-6.1%, P=0.008) between 2011 and 2017, and an APC of 6.8% (95% CI: 3.7-10.0%, P=0.004) between 2017 and 2020. Similar trend was observed for cumulative clustering rate in Wuchang as well, with an AAPC of 6.7% (95% CI: 3.2-10.3%, P<0.001) between 2009 and 2020. Despite that the increase in cumulative clustering rate during 2009-2014 period in Wuchang was significant with an APC of 11.8% (95% CI: 3.8-20.4%, P=0.004), the increase trends in cumulative clustering rate in Wuchang in 2014-2018 and 2018-2020 periods were not statistically significant.

1. Kim HJ, Fay MP, Feuer EJ, et al. Permutation tests for joinpoint regression with applications to cancer rates. Stat Med. 2000 Feb 15;19(3):335-51.

**Table S1**. Drug-resistance profile for 14 anti-TB drugs in Wusheng and Wuchang, stratified by new and retreated cases.

|  |  | **Wusheng** | | | **Wuchang** | | |
| --- | --- | --- | --- | --- | --- | --- | --- |
| **Drug** | **Drug resistance mutations** | **New cases**  **(N = 1197)** | **Retreated cases (N = 92)** | **Total**  **(N = 1289)** | **New cases**  **(N = 642)** | **Retreated cases (N = 57)** | **Total**  **(N = 699)** |
| INH | Without | 1084 (90.6) | 70 (76.1) | 1154 (89.5) | 575 (89.6) | 47 (82.5) | 622 (89.0) |
|  | With | 113 (9.4) | 22 (23.9) | 135 (10.5) | 67 (10.4) | 10 (17.5） | 77 (11.0) |
| RIF | Without | 1128 (94.2) | 73 (79.3) | 1201 (93.2) | 592 (92.2） | 43 (75.4) | 635 (90.8) |
|  | With | 69 (5.8) | 19 (20.7) | 88 (6.8) | 50 (7.8) | 14 (24.6) | 64 (9.2) |
| EMB | Without | 1158 (96.7) | 77 (83.7) | 1235 (95.8) | 627 (97.7) | 50 (87.7) | 677 (96.9) |
|  | With | 39 (3.3) | 15 (16.3) | 54 (4.2) | 15 (2.3) | 7 (12.3) | 22 (3.1) |
| PZA | Without | 1177 (98.3) | 87 (94.6) | 1264 (98.1) | 638 (99.4) | 53 (93.0) | 691 (98.9) |
|  | With | 20 (1.7) | 5 (5.4) | 25 (1.9) | 4 (0.6) | 4 (7.0） | 8 (1.1） |
| SM | Without | 1111 (92.8) | 80 (87.0) | 1191 (92.4) | 534 (83.2) | 44 (77.2) | 578 (82.7) |
|  | With | 86 (7.2) | 12 (13.0) | 98 (7.6) | 108 (16.8) | 13 (22.8) | 121 (17.3) |
| AMK | Without | 1179 (98.5) | 88 (95.7) | 1267 (98.3) | 623 (97.0) | 54 (94.7) | 677 (96.9) |
|  | With | 18 (1.5) | 4 (4.3) | 22 (1.7) | 19 (3.0) | 3 (5.3) | 22 (3.1) |
| ETO | Without | 1174 (98.1) | 88 (95.7) | 1262 (97.9) | 622 (96.9) | 57 (100.0) | 679 (97.1） |
|  | With | 23 (1.9) | 4 (4.3) | 27 (2.1) | 20 (3.1) | 0 (0.0) | 20 (2.9) |
| FQ | Without | 1161 (97.0) | 88 (95.7) | 1249 (96.9) | 614 (95.6) | 53 (93.0) | 667 (95.4) |
|  | With | 36 (3.0) | 4 (4.3) | 40 (3.1) | 28 (4.4) | 4 (7.0) | 32 (4.6) |
| KAN | Without | 1185 (99.0) | 90 (97.8) | 1275 (98.9) | 639 (99.5) | 57 (100.0) | 696 (99.6) |
|  | With | 12 (1.0) | 2 (2.2) | 14 (1.1) | 3 (0.5) | 0 (0.0) | 3 (0.4) |
| CPM | Without | 1186 (99.1) | 90 (97.8) | 1276 (99.0) | 638 (99.4) | 57 (100.0) | 695 (99.4) |
|  | With | 11 (0.9) | 2 (2.2) | 13 (1.0) | 4 (0.6) | 0 (0) | 4 (0.6) |
| PAS | Without | 1176 (98.2) | 89 (96.7) | 1265 (98.1) | 638 (99.4) | 56 (98.2) | 694 (99.3) |
|  | With | 21 (1.8) | 3 (3.3) | 24 (1.9) | 4 (0.6) | 1 (1.8) | 5 (0.7) |
| LZD | Without | 1197 (100.0) | 92 (100.0) | 1289 (100.0) | 642 (100.0) | 57 (0.0) | 699 (100.0) |
|  | With | 0 (0.0) | 0 (0.0) | 0 (0.0) | 0 (0.0) | 0 (0.0) | 0 (0.0) |
| CFZ | Without | 1197 (100.0) | 92 (100.0) | 1289 (100.0) | 641 (99.8) | 57 (100.0) | 698 (99.9) |
|  | With | 0 (0.0) | 0 (0.0) | 0 (0.0) | 1 (0.2) | 0 (0) | 1 (0.1) |
| BDQ | Without | 1197 (100.0) | 92 (100.0) | 1289 (100.0) | 641 (99.8) | 57 (100.0) | 698 (99.9) |
|  | With | 0 (0.0) | 0 (0.0) | 0 (0.0) | 1 (0.2) | 0 (0) | 1 (0.1) |

INH, Isoniazid; RIF, rifampicin; EMB, ethambutol; PZA, pyrazinamide; SM, streptomycin; AMK, amikacin; ETO, ethionamide; FQ, fluoroquinolone; KAN, kanamycin; CPM, capreomycin; PAS, para-aminosalisylic acid; LZD, linezolid; CFZ, clofazimine; BDQ, bedaquiline.

**Table S2.** Characteristics of MDR and non-MDR in Wusheng and Wuchang.

|  | **MDR (N = 102)** | **Non-MDR (N = 1886)** | **P value** |
| --- | --- | --- | --- |
| Sex |  |  | 0.679 |
| Female | 26 (25.5) | 447 (23.7) |  |
| Male | 76 (74.5) | 1439 (76.3) |  |
| Age | 47 (35, 57) | 50 (34, 62) | 0.113^a^ |
| Occupation |  |  | 0.282 |
| Farmer | 86 (84.3) | 1588 (84.2) |  |
| Students | 3 (2.9) | 115 (6.1) |  |
| Others | 13 (12.7) | 183 (9.7) |  |
| History of tuberculosis | |  | <0.001 |
| New | 78 (76.5) | 1761 (93.4) |  |
| Retreated | 24 (23.5) | 125 (6.6) |  |
| Diagnostic delay |  |  | 0.315 |
| <2 weeks | 21 (20.6) | 510 (27.0) |  |
| 2-4 weeks | 26 (25.5) | 363 (19.2) |  |
| 4-8 weeks | 31 (30.4) | 548 (29.1) |  |
| ≥8 weeks | 24 (23.5) | 465 (24.7) |  |
| Chest cavitation |  |  | 0.594 |
| No | 64 (62.7) | 1232 (65.3) |  |
| Yes | 38 (37.3) | 654 (34.7) |  |
| Sputum smear status | |  | 0.407 |
| Negative | 41 (40.2) | 837 (44.4) |  |
| Positive | 61 (59.8) | 1049 (55.6) |  |

^a^ Wilcoxon non-parametric rank sum test.

**Table S3.** Characteristics of genomic clusters with confirmed (n=110) or probable (n=15) epidemiological links based on whole-genome sequencing analysis in Wusheng.

|  | **Number of cases in clusters** | **Mean age (years [IQR])** | **Number of patients living in the same village** | **Number of new cases** | **Number of patients receiving investigation** | **Epidemiologically linked** | **Nature of epidemiological link [number of patients]^a^** |
| --- | --- | --- | --- | --- | --- | --- | --- |
| Cluster01 | 2 | 55 | 2 | 1 | 2 | Confirmed | family [2] |
| Cluster02 | 3 | 45 | 2 | 3 | 3 | Confirmed | family [2] |
| Cluster04 | 2 | 23 | 2 | 2 | 2 | Probable | same village [2] |
| Cluster10 | 3 | 47 | 3 | 3 | 3 | Confirmed/Probable | family [2]; same village [1] |
| Cluster14 | 5 | 57 | 0 | 3 | 5 | Confirmed | teahouse [5] |
| Cluster16 | 4 | 50 | 2 | 4 | 3 | Confirmed | family [2] |
| Cluster20 | 2 | 17 | 0 | 2 | 2 | Confirmed | classmate [2] |
| Cluster22 | 3 | 60 | 2 | 3 | 3 | Confirmed | family [2] |
| Cluster24 | 2 | 41 | 2 | 2 | 2 | Confirmed | family [2] |
| Cluster27 | 5 | 47 | 2 | 5 | 5 | Confirmed/Probable | workplace [3]; relatives [1]; same village [1] |
| Cluster32 | 3 | 17 | 0 | 3 | 3 | Confirmed | classmate [2] |
| Cluster35 | 3 | 18 | 0 | 3 | 3 | Confirmed | classmate [3] |
| Cluster36 | 2 | 56 | 2 | 2 | 2 | Confirmed | same village and knew each other [2] |
| Cluster37 | 2 | 18 | 0 | 2 | 2 | Confirmed | classmate [2] |
| Cluster39 | 3 | 39 | 2 | 3 | 3 | Confirmed | family [2] |
| Cluster42 | 2 | 60 | 2 | 2 | 2 | Confirmed | same village and knew each other [2] |
| Cluster44 | 7 | 54 | 3 | 6 | 7 | Confirmed | family [2]; neighbor [1] |
| Cluster45 | 2 | 57 | 2 | 2 | 2 | Confirmed | same village and knew each other [2] |
| Cluster46 | 4 | 41 | 2 | 3 | 3 | Confirmed | same village and knew each other [2] |
| Cluster48 | 2 | 44 | 2 | 2 | 2 | Confirmed | relatives [2] |
| Cluster49 | 3 | 34 | 2 | 2 | 2 | Confirmed | family [2] |
| Cluster52 | 10 | 18 | 0 | 10 | 10 | Confirmed | classmate [9] |
| Cluster53 | 2 | 49 | 2 | 2 | 2 | Confirmed | family [2] |
| Cluster55 | 2 | 48 | 2 | 2 | 2 | Confirmed | family [2] |
| Cluster57 | 3 | 51 | 2 | 2 | 3 | Confirmed | family [2]; teacher [1] |
| Cluster61 | 3 | 59 | 2 | 3 | 3 | Confirmed | family [2] |
| Cluster66 | 2 | 31 | 2 | 2 | 2 | Confirmed | family [2] |
| Cluster67 | 9 | 32 | 3 | 8 | 8 | Confirmed | family [3]; classmate [3]; same village and knew each other [1] |
| Cluster69 | 6 | 57 | 0 | 6 | 6 | Confirmed | friends [2] |
| Cluster70 | 2 | 51 | 2 | 2 | 2 | Probable | same village [2] |
| Cluster72 | 3 | 62 | 0 | 3 | 3 | Confirmed | teahouse [2] |
| Cluster73 | 3 | 45 | 0 | 3 | 3 | Confirmed | mental hospital [3] |
| Cluster75 | 3 | 64 | 2 | 3 | 3 | Confirmed | friends [2] |
| Cluster78 | 2 | 41 | 2 | 2 | 2 | Confirmed | friends [2] |
| Cluster79 | 5 | 38 | 5 | 5 | 5 | Confirmed/Probable | family [2]; same village [3] |
| Cluster86 | 2 | 58 | 2 | 2 | 2 | Confirmed | same village and knew each other [2] |
| Cluster87 | 4 | 24 | 0 | 4 | 4 | Confirmed | classmate [3] |
| Cluster88 | 3 | 56 | 2 | 3 | 3 | Confirmed | same village and knew each other [2] |
| Cluster93 | 3 | 46 | 0 | 3 | 3 | Confirmed | mental hospital [2] |
| Cluster97 | 2 | 55 | 0 | 2 | 2 | Confirmed | friends [2] |
| Cluster98 | 2 | 50 | 2 | 2 | 2 | Probable | same village [2] |
| Cluster103 | 2 | 30 | 0 | 2 | 2 | Confirmed | friends [2] |
| Cluster107 | 7 | 32 | 6 | 6 | 7 | Confirmed/Probable | family [2]; same village [4] |
| Cluster109 | 2 | 44 | 2 | 2 | 2 | Confirmed | same village and knew each other [2] |
| Cluster111 | 2 | 34 | 2 | 2 | 2 | Confirmed | friends [2] |
| Cluster117 | 2 | 59 | 2 | 2 | 2 | Confirmed | friends [2] |
| Cluster119 | 2 | 31 | 2 | 2 | 2 | Confirmed | relatives [2] |

^a^ Red indicates household contacts, yellow indicates social contacts, black indicates genomic-clustered cases with probable epidemiological links.

**Table S4.** Characteristics of genomic clusters with confirmed (n=86) or probable (n=11) epidemiological links based on whole-genome sequencing analysis in Wuchang.

|  | **Number of cases in clusters** | **Mean age (years [IQR])** | **Number of patients living in the same village** | **Number of new cases** | **Number of patients receiving investigation** | **Epidemiologically linked** | **Nature of epidemiological link [number of patients]^a^** |
| --- | --- | --- | --- | --- | --- | --- | --- |
| Cluster03 | 5 | 52 | 3 | 5 | 4 | Confirmed | family [2]; same village and knew each other [1] |
| Cluster04 | 6 | 45 | 2 | 6 | 4 | Confirmed | same village and knew each other [2] |
| Cluster05 | 4 | 49 | 3 | 3 | 4 | Confirmed | same village and knew each other [1]; relatives [2] |
| Cluster08 | 3 | 71 | 2 | 3 | 3 | Confirmed | relatives [2] |
| Cluster09 | 2 | 19 | 0 | 2 | 2 | Confirmed | workplace [2] |
| Cluster11 | 3 | 18 | 0 | 3 | 3 | Confirmed | classmates [3] |
| Cluster12 | 8 | 43 | 4 | 7 | 7 | Confirmed | family [3]; same village and knew each other [2] |
| Cluster17 | 2 | 55 | 2 | 2 | 2 | Confirmed | same village and knew each other [2] |
| Cluster21 | 2 | 40 | 0 | 2 | 2 | Confirmed | same village and knew each other [2] |
| Cluster23 | 2 | 27 | 2 | 2 | 0 | Probable | same village [2] |
| Cluster25 | 7 | 54 | 6 | 7 | 6 | Confirmed/probable | same village and knew each other [4]; same village [2] |
| Cluster27 | 5 | 57 | 2 | 5 | 3 | Confirmed | family [2] |
| Cluster30 | 3 | 55 | 0 | 3 | 2 | Confirmed | family [2] |
| Cluster37 | 3 | 43 | 2 | 3 | 2 | Confirmed | same village and knew each other [2] |
| Cluster39 | 6 | 54 | 0 | 6 | 3 | Confirmed | Workplace [2] |
| Cluster40 | 3 | 38 | 2 | 3 | 0 | Probable | same village [2] |
| Cluster42 | 2 | 32 | 2 | 2 | 2 | Confirmed | family [2] |
| Cluster44 | 2 | 45 | 2 | 0 | 2 | Confirmed | family [2] |
| Cluster45 | 4 | 45 | 3 | 4 | 4 | Confirmed | same village and knew each other [3] |
| Cluster47 | 3 | 50 | 0 | 3 | 3 | Confirmed | nursing home [3] |
| Cluster50 | 3 | 41 | 0 | 3 | 3 | Confirmed | family [2] |
| Cluster53 | 3 | 40 | 0 | 3 | 3 | Confirmed | friends [3] |
| Cluster54 | 3 | 36 | 0 | 3 | 0 | Confirmed | family [2] |
| Cluster55 | 3 | 46 | 3 | 3 | 3 | Confirmed | same village and knew each other [3] |
| Cluster56 | 4 | 47 | 3 | 4 | 3 | Confirmed/Probable | same village and knew each other [2]; same village [1] |
| Cluster58 | 3 | 46 | 2 | 3 | 0 | Probable | same village [2] |
| Cluster60 | 12 | 43 | 6 | 11 | 10 | Confirmed | relatives [2]; same village and knew each other [6] |
| Cluster64 | 2 | 63 | 2 | 2 | 2 | Confirmed | same village and knew each other [2] |
| Cluster66 | 2 | 44 | 2 | 2 | 2 | Confirmed | same village and knew each other [2] |
| Cluster69 | 2 | 36 | 2 | 2 | 2 | Confirmed | family [2] |
| Cluster70 | 2 | 39 | 2 | 2 | 0 | Probable | same village [2] |
| Cluster73 | 8 | 50 | 0 | 6 | 6 | Confirmed | family [2] |
| Cluster77 | 3 | 53 | 2 | 3 | 2 | Confirmed | same village and knew each other [2] |
| Cluster80 | 2 | 68 | 2 | 2 | 2 | Confirmed | same village and knew each other [2] |
| Cluster82 | 2 | 33 | 2 | 2 | 2 | Confirmed | same village and knew each other [2] |
| Cluster89 | 2 | 42 | 2 | 2 | 2 | Confirmed | family [2] |
| Cluster94 | 4 | 39 | 2 | 4 | 2 | Confirmed | same village and knew each other [2] |
| Cluster95 | 2 | 41 | 2 | 2 | 2 | Confirmed | same village and knew each other [2] |

^a^ Red indicates household contacts, yellow indicates social contacts, black indicates genomic-clustered cases with probable epidemiological links.

**Figure S1.** Map of China showing the distribution of the tuberculosis patients whose isolates of *Mycobacterium tuberculosis* were included in the study. The selected study field sites were Wusheng county, Sichuan Province and Wuchang county, Heilongjiang Province. The markers on the map indicate the relative location of the 2 study sites.

**Figure S2.** Reported incidence of tuberculosis in Wusheng (left) and Wuchang (right), 2009-2020.

**Figure S3.** Changes in temporal trends in cumulative clustering rate in Wusheng (left) and Wuchang (right), 2009-2020.


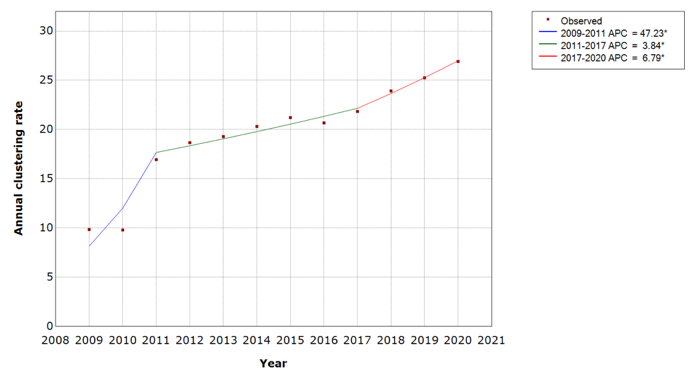

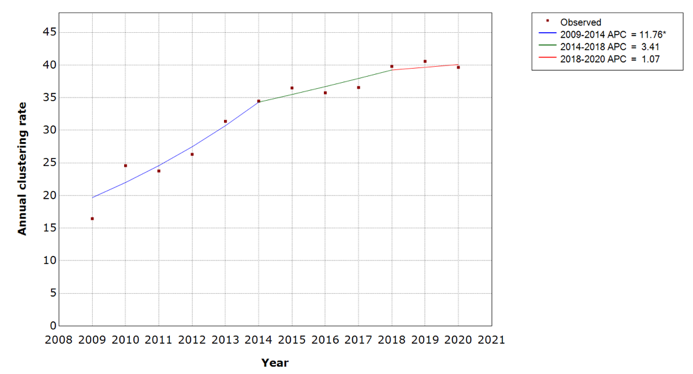


^*^APC is significantly different from zero at the level of α=0.05.

**Figure S4.** Number and proportion of culture-positive tuberculosis in Wusheng (left) and Wuchang (right), 2013-2020.

**Figure S5.** Transmission networks of multidrug-resistant tuberculosis based on genomic phylogeny and drug-resistance mutations. For each network, the arrow indicates the root and circles represent *Mycobacterium tuberculosis* isolates. *M tuberculosis* isolates are separated by lines with length representing genetic distance. Different colors of rectangle indicate resistance to different drugs. Two new MDR-TB patients were clustered with non-MDR-TB patients and were not included in the analysis.

**
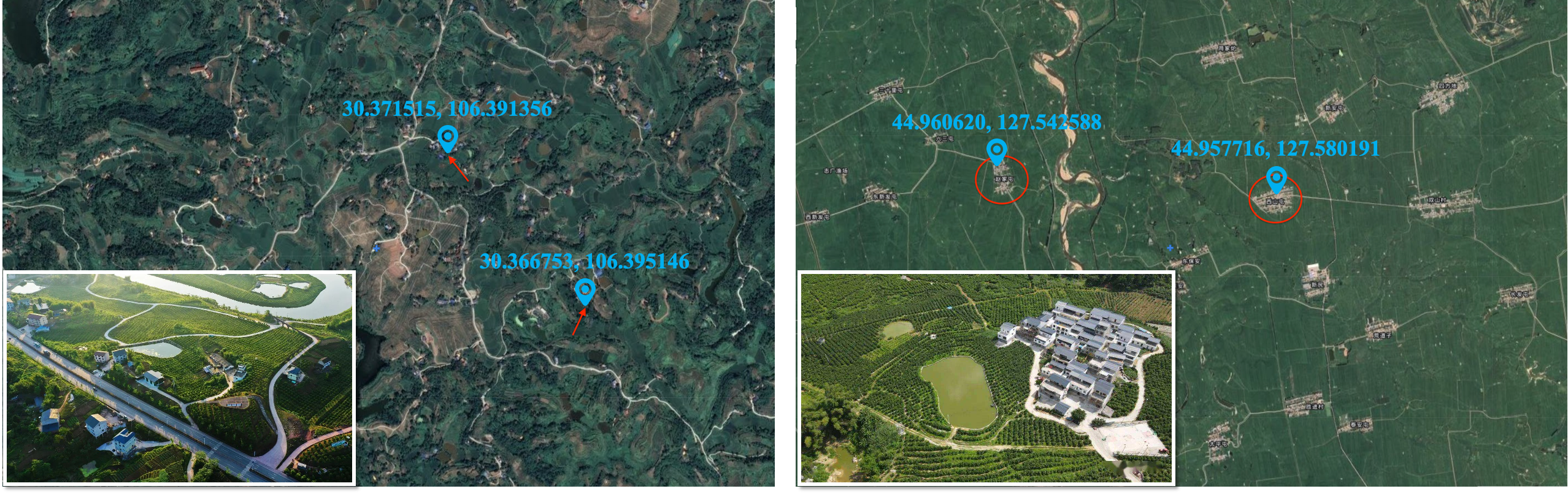
**

**Figure S6.** Residence habits in Wusheng (left) and Wuchang (right). In Wusheng, the boundaries of the village are not obvious. The houses are scattered (red arrow). People are spatially distant from each other. Only people who live close know and interact with each other. The average annual temperature in Wusheng is 18 ℃, and people spend more time outdoors. In Wuchang, the boundaries of the village are pretty obvious (red circle). The houses are adjacent to each other, and a dozen families form a small village. Almost everyone in the village knows and interacts with each other. The average annual temperature in Wuchang is only 4 ℃, and people spend more time indoors. Villagers often gather together to chat and play cards.
